# Efficient Marker-Assisted Pyramiding of *Xa21* and *Xa23* Genes into Elite Rice Restorer Lines Confers Broad-Spectrum Resistance to Bacterial Blight

**DOI:** 10.3390/plants14142107

**Published:** 2025-07-09

**Authors:** Yao Li, Yulong Fan, Yihang You, Ping Wang, Yuxuan Ling, Han Yin, Yinhua Chen, Hua Zhou, Mingrui Luo, Bing Cao, Zhihui Xia

**Affiliations:** 1 School of Breeding and Multiplication (Sanya Institute of Breeding and Multiplication), Hainan University, Sanya 572025, China; sdly1997@163.com (Y.L.); fanyulong2012@126.com (Y.F.); 15532122152@163.com (Y.Y.); 15020362601@163.com (P.W.); lingyuxuan2021@163.com (Y.L.); 18360574102@163.com (H.Y.); yhchen@hainanu.edu.cn (Y.C.); 2 Life Science and Technology Center, China National Seed Group Co., Ltd., Wuhan 430206, China; hua.zhou@syngentagroup.cn (H.Z.); mingrui.luo@syngentagroup.cn (M.L.)

**Keywords:** bacterial blight, *Xa21*, *Xa23*, pyramiding, backcross, rice, functional markers, breeding

## Abstract

Bacterial blight (BB) caused by *Xanthomonas oryzae* pv. *oryzae* (*Xoo*) is a major threat to global rice productivity. Although hybrid rice breeding has significantly enhanced yields, persistent genetic vulnerabilities within restorer lines continue to compromise BB resistance. This study addresses this challenge by implementing functional marker-assisted selection (FMAS) to pyramid two broad-spectrum resistance (R) genes, *Xa21* and *Xa23*, into the elite, yet BB-susceptible, restorer line K608R. To enable precise *Xa23* genotyping, we developed a novel three-primer functional marker (FM) system (IB23/CB23/IR23). This system complements the established U1/I2 markers used for *Xa21*. This recombination-independent FMAS platform facilitates simultaneous, high-precision tracking of both homozygous and heterozygous alleles, thereby effectively circumventing the linkage drag limitations typical of conventional markers. Through six generations of marker-assisted backcrossing followed by intercrossing, we generated K608R2123 pyramided lines harboring both R genes in homozygous states, achieving a recurrent parent genome recovery rate of 96.93%, as determined by single nucleotide polymorphism (SNP) chip analysis. The pyramided lines exhibited enhanced resistance against six virulent *Xoo* pathogenic races while retaining parental yield performance across key agronomic traits. Our FMAS strategy overcomes the historical trade-off between broad-spectrum resistance and the preservation of elite phenotypes, with the developed lines exhibiting resistance coverage complementary to that of both introgressed R genes. This integrated approach provides breeders with a reliable molecular tool to accelerate the development of high-yielding, disease-resistant varieties, demonstrating significant potential for practical deployment in rice improvement programs. The K608R2123 germplasm represents a dual-purpose resource suitable for both commercial hybrid seed production and marker-assisted breeding programs, and it confers synergistic resistance against diverse *Xoo* races, thereby providing a pivotal breeding resource for sustainable BB control in epidemic regions.

## 1. Introduction

Rice (*Oryza sativa* L.), a staple crop sustaining global food security, accounts for approximately 50% of global dietary consumption. *Xanthomonas oryzae* pv. *oryzae* (*Xoo*) is a Gram-negative bacterial genus in the family Xanthomonadaceae that causes many important diseases in a variety of plant hosts, such as rice, wheat, citrus, tomato, and pepper [1]. Bacterial blight (BB) caused by *Xoo* ranks among the most devastating diseases in rice cultivation, leading to significant reductions in both grain yield and quality [2]. *Xoo* typically enters rice plants via water pores or wounds, subsequently multiplying within vascular bundles and causing blockage. The disease initially manifests at the leaf tip, followed by progressive yellowing that spreads throughout the leaf, ultimately leading to leaf death [3]. In China, hybrid rice cultivars, which occupy more than 50% of the total cultivated rice area, exhibit widespread susceptibility to BB [4]. Globally, BB contributes to substantial yield losses in hybrid rice production due to the susceptibility of parental lines to virulent *Xoo* races prevalent in field conditions [5].

Conventional approaches, such as chemical control, often prove ineffective in enhancing BB resistance in rice. Empirical studies indicate that developing elite parental lines with pyramided BB resistance represents the most cost-effective, efficient, and environmentally sustainable strategy in hybrid rice breeding programs. Within the three-line hybrid breeding system—comprising a cytoplasmic male sterile (CMS) line (female parent), maintainer line, and restorer line (male parent)—the integration of BB resistance (R) genes into restorer lines is critical for fortifying genetic resistance in hybrid progeny [6]. However, rapid pathogen evolution drives continuous diversification of *Xoo* races, eroding monogenic resistance in field-deployed germplasm [7]. A prominent example is the diminished efficacy of *Xa4* in restorer lines following prolonged cultivation [8]. Furthermore, given the limited spectrum of individual R genes, pyramiding multiple BB resistance genes into restorer lines has emerged as a pivotal objective in hybrid rice breeding. To date, 44 BB resistance genes have been identified and genetically mapped in rice [9]. The cloned *Xa21* gene confers broad-spectrum resistance against diverse *Xoo* races, including eight predominant strains in China [10,11]. *Xa21* encodes a receptor-like kinase characterized by three functional domains: a leucine-rich repeat (LRR) motif, a transmembrane helix, and an intracellular kinase domain [12,13]. Similarly, *Xa23* confers resistance to over 20 *Xoo* races [14]. The near-isogenic line CBB23 was obtained by transferring *Xa23* from wild rice into the susceptible cultivar JG30, which exhibits broad-spectrum resistance to *Xoo* [15]. The complementary resistance spectra of *Xa21* and *Xa23* confer broad-spectrum and durable resistance against BB, while their co-localization within a tightly linked region on chromosome 11 provides a distinct practical advantage; it enables both genes to be introgressed and inherited as a single genetic block. Consequently, breeding lines pyramiding these genes not only acquire enhanced BB resistance but also serve as efficient donor sources for co-transferring both resistance traits into future varieties. Guided by this dual rationale of functional synergy and genetic linkage, in the current study, *Xa21* and *Xa23* were selected for pyramiding into the elite restorer line K608R.

An ideal pyramiding product should contain as little donor genome as possible in the genetic background of an elite recurrent variety. Recurrent backcrossing was a traditional breeding method employed to transfer alleles from a donor to an elite cultivar. However, in breeding for rice disease resistance, the temporal expression patterns of resistance genes pose unique challenges for phenotypic selection [16]. For instance, while *Xa23* confers resistance across all developmental stages [17], *Xa21* exhibits age-dependent resistance, functioning primarily in plants older than six weeks [18,19]. This temporal disparity renders seedling-stage resistance screening ineffective for selecting pyramided plants containing both *Xa21* and *Xa23*. Marker-assisted selection (MAS) provides an effective tool for tracking the introgression of resistance genes from donor parents into susceptible cultivars during breeding cycles [20,21]. Previous studies have validated the utility of MAS for gene pyramiding in rice [22,23,24,25,26]. Therefore, in this way, based on the combination of backcrosses and MAS, it is possible to track the specific resistance loci in segregated populations while avoiding phenotypic screening [27], and to recover the recurrent parent genome in less time in comparison to conventional breeding [28].

Conventional MAS typically relies on random DNA markers derived from genomic regions linked to target loci. A critical limitation of such markers is their dependence on conserved linkage phases between markers and target loci [29]. Linkage drag and recombination events during successive breeding generations can disrupt marker–trait associations, particularly for distally located markers, thereby compromising selection accuracy. By contrast, functional markers (FMs) are derived from polymorphic sites within genes that causally affect phenotypic trait variation. Once genetic effects have been assigned to functional sequence motifs, FMs can be used to fix gene alleles across several genetic backgrounds without additional calibration [30]. Thus, these limitations have driven the adoption of FMs, which target causal polymorphisms within gene sequences, enabling precision MAS [31].

In summary, this study considered the following: (1) overcoming precision limitations of conventional MAS by eliminating recombination-dependent genotyping errors during pyramiding of closely linked resistance genes (*Xa21*/*Xa23* on chromosome 11); (2) resolving susceptibility of hybrid rice restorer lines to evolving *Xoo* races; and (3) breaking the historical trade-off between enhanced BB resistance and yield potential. To address these three critical challenges, we developed a novel three-primer FMs system (IB23/CB23/IR23) for *Xa23* to complement the established U1/I2 markers for *Xa21* designed by Xia et al. [32]. This integrated functional marker-assisted selection (FMAS) platform enables recombination-independent allele tracking and precise discrimination of both loci. The resultant K608R2123 lines pyramided *Xa21* and *Xa23* in homozygous states, with SNP analysis confirming 96.93% recurrent parent genome recovery. Crucially, K608R2123 exhibited enhanced resistance against six *Xoo* pathogenic races, including the prevalent Hainan race XOO4, surpassing parental resistance levels while maintaining yield performance comparable to the elite restorer K608R.

## 2. Results

### 2.1. Functional Marker Development

**Figure 1:**
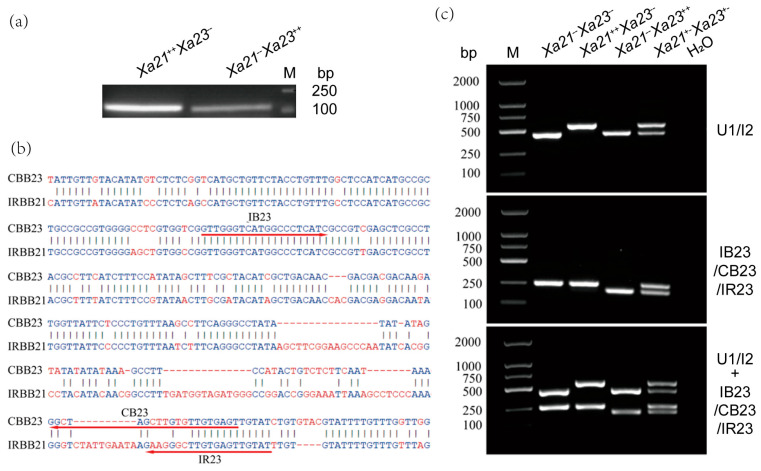
Functional marker development. (**a**) PCR amplification of *Xa21*^++^*Xa23*^−−^ and *Xa21*^−−^*Xa23*^++^ was performed using primers RM206. M: DL2000 DNA ladder marker. (**b**) Schematic diagram of *Xa23* functional marker, the arrow shows the sequence and position of detection primers. (**c**) PCR amplification of *Xa21*^−−^*Xa23*^−−^, *Xa21*^++^*Xa23*^−−^, *Xa21*^−−^*Xa23*^++^ and *Xa21*^+−^*Xa23*^+−^ was performed using primers U1/I2, IB23/CB23/IR23. M: DL2000 DNA ladder marker. H_2_O: Use water as a negative control.

*Xa21* and *Xa23* genes were introgressed into K608R through crossing with IRBB21 (*Xa21* donor) and CBB23 (*Xa23* donor). We developed precision molecular markers for pyramiding *Xa21* and *Xa23* following these introgressions. The U1/I2 codominant marker system [32] was employed for precise discrimination of *Xa21* genotypes, generating distinct 574 bp (homozygous *Xa21*^++^), 447 bp (null *Xa21*^−−^), and dual-band (heterozygous *Xa21*^+−^) amplification profiles (Figure 1c). In the context of resolution limitations in *Xa23* genotyping, conventional RM206 marker analysis revealed insufficient electrophoretic discrimination between 127 bp (*Xa23*^++^) and 119 bp (*Xa23*^−−^) amplicons (Figure 1a). We developed a tri-primer system (IB23/CB23/IR23) targeting sequence polymorphisms between CBB23 (*Xa23* donor) and IRBB21 (*Xa21* donor, recurrent parent, IR24 genetic background). This system employed the following components: (1) IB23: universal forward primer; (2) CB23: *Xa23*-specific reverse primer; (3) IR23: susceptible allele-specific reverse primer. The optimized configuration yielded differential amplification: 194 bp for *Xa23*^++^ versus 251 bp for *Xa23*^−−^, with heterozygous plants exhibiting both bands (Figure 1b). Compared with RM206’s 8 bp fragment difference, the tri-primer system achieved a resolvable 57 bp size differentiation, enabling unambiguous genotype calls. Multiplex integration of U1/I2 and IB23/CB23/IR23 markers permitted simultaneous *Xa21*/*Xa23* detection in single PCR reactions. The combined system generated four diagnostic patterns: *Xa21*^++^/*Xa23*^++^ (574 + 194 bp); *Xa21*^+−^/*Xa23*^+−^ (574/447 + 194/251 bp); *Xa21*^−−^/*Xa23*^−−^ (447 + 251 bp); and hetero-combinations (corresponding mixed bands) (Figure 1c). Notably, the IR23 primer was designed based on IRBB21/IR24 sequence homology at the recessive *Xa23* locus, ensuring reliable performance in IR24-background varieties (Figure 1b).

### 2.2. Xa21/Xa23 Pyramiding via MAS

**Figure 2:**
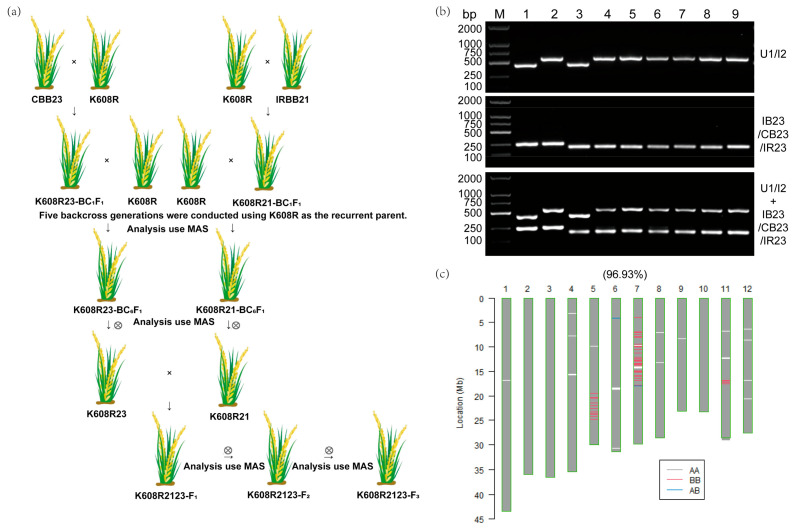
*Xa21*/*Xa23* Pyramiding via MAS. (**a**) A roadmap for developing rice restorer lines with broad-spectrum resistance to bacterial blight. (**b**) PCR amplification of K608R (1), K608R21 (2), K608R23 (3), and K608R2123 (4–9) was performed using primers U1/I2, IB23/CB23/IR23. M: DL2000 DNA ladder marker. (**c**) Genetic background purity of K608R2123.

We selected K608R as the recurrent parent, with CBB23 (carrying *Xa23*) and IRBB21 (containing *Xa21*) as donor parents. To simultaneously introgress the target R genes and maintain the agronomic traits of K608R, we generated six successive backcrosses (BC_1_–BC_6_) with each donor parent, integrating phenotypic selection for recurrent parent characteristics with MAS. For precise genotype verification, we applied gene-specific markers: IB23/CB23/IR23 markers to detect *Xa23* in the derived lines, and U1/I2 markers to identify *Xa21*. This process ultimately yielded BC_6_F_1_ plants designated as K608R21-BC6F1 (carrying *Xa21*) and K608R23-BC6F1 (carrying *Xa23*). We subsequently self-pollinated these BC_6_F_1_ lines and performed MAS screening to obtain homozygous progenies: the *Xa21*-homozygous line K608R21 and the *Xa23*-homozygous line K608R23. To achieve gene pyramiding, we hybridized K608R21 with K608R23 to produce F_1_ hybrids (K608R2123-F_1_) (Figure 2a). Two consecutive selfing generations (F_2_ and F_3_) were then raised to ensure genetic stabilization. In the F_2_ population comprising 624 plants, we identified three chimeric plants with *Xa21*^+−^*Xa23*^++^ genotypes and two with *Xa21*^++^*Xa23*^+−^ genotypes, corresponding to a recombination rate of 0.84% (Table 1). Through MAS-based selection, we successfully developed the homozygous pyramided line K608R2123, which stably harbors both *Xa23* and *Xa21* in homozygous states (Figure 2b). Finally, genome-wide SNP analysis using the Rice120K array demonstrated that K608R2123 exhibited 96.93% background recovery relative to the recurrent parent K608R (Figure 2c).

### 2.3. Broad-Spectrum Resistance Evaluation

**Figure 3:**
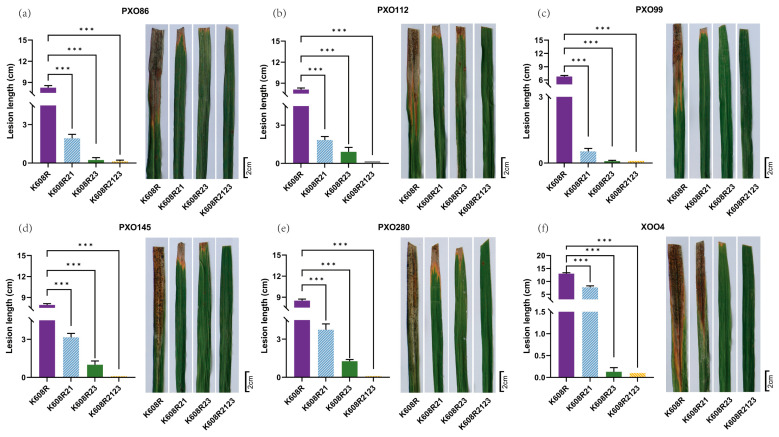
Evaluation of Broad-Spectrum BB Resistance. BB resistance in K608R, K60821, K608R23, and K608R2123, 14 days post-inoculation with six different physiological strains of *Xoo* ((**a**) PXO86, (**b**) PXO112, (**c**) PXO99, (**d**) PXO145, (**e**) PXO280, and (**f**) XOO4) using the leaf clipping method. Bar = 2 cm. Data represent means + standard deviations. The asterisks indicate significant differences between K608R and the plants containing *Xa21* (K608R21), the plants containing *Xa23* (K608R23), and the plants with pyramided *Xa21* and *Xa23* (K608R2123) (*** *p* < 0.001, *n* = 10). *p* values calculated by Student’s two-tailed *t*-test.

We investigated the broad-spectrum resistance of the pyramided line K608R2123 against BB, using the recurrent parent K608R and two monogenic lines (K608R21 and K608R23) as controls. At the tillering stage, plants were inoculated under field conditions with six distinct *Xoo* races. Disease resistance was evaluated 14 days post-inoculation by measuring lesion lengths. The K608R2123 line, harboring both *Xa21* and *Xa23* resistance genes, exhibited significantly shorter lesion lengths compared to the parental controls (K608R, K608R21, and K608R23). Notably, K608R2123 demonstrated enhanced resistance against all six *Xoo* races tested (Figure 3). Furthermore, the pyramided line displayed expanded resistance spectra relative to its parental lines. Additionally, to preliminarily assess resistance persistence, we conducted a supplemental validation at the same Hainan field station in 2025. When challenged with the dominant XOO4 strain (Figure S1), K608R2123 maintained significantly stronger resistance than the parental lines. These results conclusively show that the gene-pyramiding strategy substantially improves both resistance intensity and pathogen coverage in rice BB defense.

### 2.4. Agronomic Performance and Yield Stability

**Figure 4:**
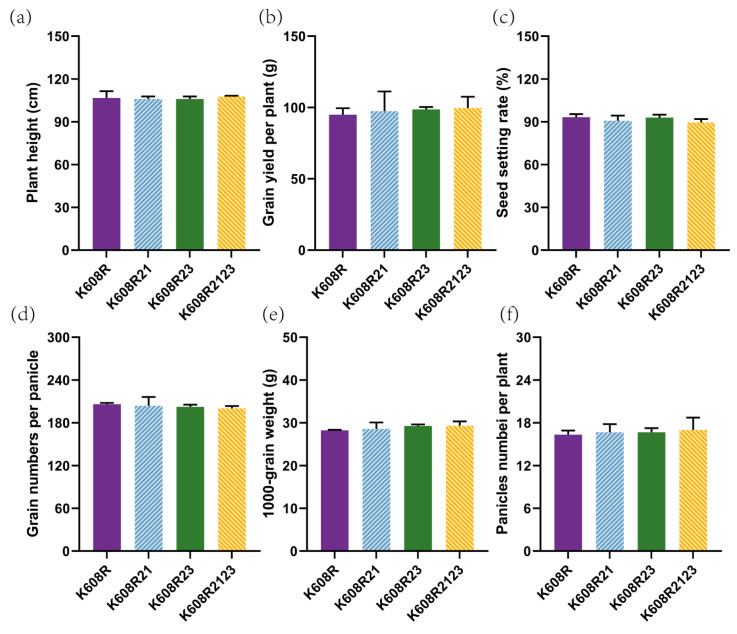
Agronomic Performance and Yield Stability. (**a**) Plant height of K608R, K60821, K608R23, and K608R2123. (**b**) Grain yield per plant of K608R, K60821, K608R23, and K608R2123. (**c**) Seed setting rate of K608R, K60821, K608R23, and K608R2123. (**d**) Grain numbers per panicle of K608R, K60821, K608R23, and K608R2123. (**e**) Thousand-grain weight of K608R, K60821, K608R23, and K608R2123. (**f**) Panicle numbers per plant of K608R, K60821, K608R23, and K608R2123. Data represent mean standard deviations, *n* = 3. *p* values according to Student’s two-tailed *t*-test. An asterisk indicates statistical significance, while the absence of an asterisk denotes no significant difference.

We conducted systematic comparisons of yield-related traits between the pyramided line K608R2123 and its parental lines (K608R, K608R21, K608R23). No significant differences (*p* > 0.05, ANOVA) were observed in seven critical agronomic parameters: plant height, panicle length, panicles per plant, panicle weight, grain number per panicle, 1000-grain weight, and yield per plant between K608R2123 and its parental lines (K608R, K608R21, K608R23) (Figure 4). All evaluated traits exhibited coefficients of variation (CVs) below 10% across three experimental replicates (Table 2), confirming the phenotypic stability of the pyramided line. These results collectively demonstrate that the *Xa21*/*Xa23* pyramiding process maintained the elite agronomic profile of the parental line while achieving enhanced disease resistance.

## 3. Discussion

The development of rice varieties with durable broad-spectrum resistance to BB remains a critical objective in modern breeding programs. While hybrid rice technology has significantly increased yields, many popular hybrids remain susceptible to BB due to parental line vulnerability, causing substantial yield losses globally [5,6]. In this study, we successfully pyramided two major BB resistance genes, *Xa21* and *Xa23*, into the elite restorer line K608R, using a FMAS approach. The resultant pyramided line, K608R2123, exhibits enhanced resistance against multiple *Xoo* races while retaining the agronomic performance of the recurrent parent. Our findings underscore the effectiveness of FMAS in accelerating gene pyramiding and minimizing linkage drag, offering a robust strategy for improving disease resistance in hybrid rice breeding.

Conventional MAS, though widely adopted, faces limitations due to recombination between linked markers and target genes, as well as an inability to distinguish functional alleles. Random DNA markers suffer from reduced accuracy across generations due to linkage phase breakdown [29]. In contrast, FMs target causal polymorphisms, eliminating recombination dependency and bypassing the need for linkage mapping [31]. Here, we overcame traditional MAS constraints by deploying FMs specific to the functional polymorphisms of *Xa21* and *Xa23*. The established U1/I2 system [32] enabled precise discrimination of the *Xa21* allele, while our novel tri-primer FM (IB23/CB23/IR23) resolved prior ambiguities in *Xa23* genotyping. Integration of these FMs into a multiplex PCR platform permitted simultaneous recombination-independent tracking of both loci, enhancing selection accuracy. The high recurrent parent genome recovery (96.93%) in K608R2123 underscores FMAS’s efficacy in minimizing donor segment retention, which is a persistent challenge in backcross breeding [28]. This rapid background recovery was facilitated by stringent phenotypic selection for recurrent parent traits in each generation, contrasting with conventional methods hindered by time-consuming multi-gene transfer [33]. Our results align with studies confirming FMAS accelerates near-isogenic line development while reducing linkage drag [31].

Single-gene resistance in rice is often vulnerable to breakdown due to the rapid evolution and differentiation of *Xoo* races [7], and individual R genes typically confer limited impacts. Therefore, pyramiding multiple BB R genes into restorer lines is a critical strategy for achieving broad-spectrum resistance in hybrid breeding [6]. Our study demonstrates that pyramiding *Xa21* and *Xa23* provides broader and enhanced resistance compared with monogenic lines. The K608R2123 line exhibited significantly shorter lesion lengths against all six tested *Xoo* races, indicating a synergistic interaction through combining these two resistance genes. Notably, the XOO4 race, which is predominant in Hainan Province, a critical region for rice breeding and seed production (“nanfan”) in China, was effectively controlled by K608R2123. This resistance underscores the line’s strong potential for application in southern China’s rice breeding programs, particularly in Hainan’s winter nursery system where the threat of bacterial blight is severe.

It is likely that complementary resistance mechanisms underpin this enhanced protection. *Xa21* encodes a receptor-like kinase that recognizes pathogen-associated molecular patterns [12], providing broad-spectrum resistance against diverse strains, including eight that are prevalent in China [11]. Furthermore, *Xa21* not only inhibits *Xoo* growth but also induces extensive disturbance of the rice transcriptome and mediates signal pathways, preventing infection [34]. *Xa23* is recognized as one of the broadest-spectrum R genes against BB, conferring resistance to numerous *Xoo* races [19,35,36], and it is widely used in breeding. Therefore, the pyramiding of *Xa21* and *Xa23* underpins the high level of broad-spectrum resistance observed in K608R2123. This complementary action probably contributed to the particularly broad-spectrum resistance observed in the two-gene pyramid line. The physical proximity of both genes on chromosome 11 facilitated their simultaneous introgression; however, the low recombination rate (0.84% in our F_2_ population) observed in the F_2_ population indicates tight linkage, necessitating precise genotyping to avoid selection bias. The broad-spectrum resistance of K608R2123 strongly supports the strategy of stacking multiple R genes to counteract pathogen diversity.

A major concern in resistance breeding is the potential yield penalty associated with introgressing disease resistance genes [36] and the risk of co-introducing undesirable traits, which increases with the number of genes that are introgressed [37]. However, our agronomic evaluations revealed no significant differences between K608R2123 and the recurrent parent K608R across key yield-related traits, including plant height, panicle characteristics, seed-setting rate, single plant yield, and grain yield. This confirms that the pyramiding process did not disrupt the elite genetic background of K608R, making K608R2123 a suitable parental line for hybrid rice production.

Given that restorer lines play a pivotal role in determining the resistance and yield potential of hybrid rice [6], the development of K608R2123 provides a valuable genetic resource for breeding BB-resistant hybrids. The stability of resistance and yield performance under field conditions suggests that this line could be deployed in regions where BB is a major constraint, particularly in China, where hybrid rice dominates cultivation [4]. Its demonstrated resistance against the Hainan-prevalent XOO4 race further positions K608R2123 as an ideal candidate for southern China’s breeding programs and “nanfan” seed production systems. Furthermore, due to their proximity on chromosome 11, *Xa21* and *Xa23* can be collectively inherited, establishing K608R2123 as a donor for disease-resistant haplotypes.

While this study demonstrates the efficacy of FMAS in pyramiding *Xa21* and *Xa23*, several limitations warrant acknowledgment. First, although tight linkage between *Xa21* and *Xa23* on chromosome 11 facilitates co-introgression, it reduces recombination rates for other beneficial genes on this chromosome, particularly those located between the two target loci. Second, the validation of agronomic traits was conducted under controlled field conditions; consequently, yield stability across diverse disease pressures and soil environments requires evaluation through multi-environment trials. Additionally, while FMAS significantly reduces linkage drag around target loci, residual donor segments beyond the selected region may persist. Such segments could subtly influence minor traits (e.g., tiller angle or grain chalkiness), necessitating future purification via high-density genotyping. Finally, the FMAS protocol requires further optimization to achieve cost-effective implementation in resource-limited breeding programs.

## 4. Materials and Methods

### 4.1. Plant Material

The rice blast nursery investigation was performed during April to May in two consecutive years (2021–2024). The rice restorer line K608R served as the recurrent parent. The donor parents were IRBB21, harboring the *Xa21* resistance gene [10,11,12,13], and CBB23, harboring the *Xa23* resistance gene [14,15]. All plant materials were provided by our laboratory. The rice seeds were surface sterilized by washing in 70% ethanol for 1 min, followed by treatment with 30% sodium hypochlorite solution for 30 min. Subsequently, the seeds were rinsed three times with sterile distilled water for 5 min each. The sterilized seeds were then soaked in distilled water and germinated in the dark at 37 °C for 48 h each (soaking and germination). Germinated seeds exhibiting emerged radicles were sown in seedling trays filled with a mixture of nutrient soil and vermiculite (3:1, *v*/*v*). Seedlings were transplanted after reaching the 3- to 4-leaf stage. Row spacing of conventionally planted plants (16.6 cm × 23.3 cm) was used, and conventional field management was conducted. Field trials were conducted during the rice-growing seasons of 2021–2024 at experimental stations (November–April) in the Batou base of Hainan University in Sanya, Hainan Province (18°21′ N, 109°10′ E), China.

### 4.2. Molecular Marker Development and Analysis

The primer sequences used in this study were as follows. *Xa21* genotyping was performed using primers U1 (5′-CGATCGGTATAACAGCAAAAC-3′) and I2 (5′-TCTGATCATGCATGTTCTGTG-3′) designed by Xia et al. [32]. For *Xa23* genotyping, the conventional codominant marker RM206 (F: 5′-CCCATGCGTTTAACTATTCT-3′, R: 5′-CGTTCCATCGATCCGTATGG-3′) was employed. To develop an improved codominant diagnostic marker for *Xa23*, high-throughput sequencing was performed to obtain the genomic sequences of the *Xa23* allele in rice variety IR24 and the *Xa23* allele in CBB23. Polymorphic sites between these alleles were identified, enabling the design of a novel tri-primer marker system: IB23 (5′-GTTGGGTCATGGCCCTCATC-3′), CB23 (5′-ACTCACAACACAAGCTAGCC-3′), and IR23 (5′-ATACAACTCACAAGCCCTTC-3′). This optimized system was used for *Xa23* genotyping in subsequent analyses. The leaf samples were collected from the field in liquid nitrogen and DNA extraction was carried out with modifications to the protocol by Murray and Thompson [38]. Genotyping of *Xa21* and *Xa23* was performed via PCR amplification in 20 μL reaction volumes containing 2 μL genomic DNA template, 0.5 μL of each relevant primer (for the spec ific marker system), and 10 μL of 2× Rapid Taq Master Mix (Vazyme, Nanjing, China). The thermal cycling protocol comprised initial denaturation at 94 °C for 4 min, followed by 35 amplification cycles each consisting of denaturation at 94 °C for 30 s, annealing at 57 °C for 30 s, and extension at 72 °C for 40 s, and subsequently, final extension at 72 °C for 10 min.

### 4.3. Assessment of Background Recovery

The Rice120k SNP array (China National Seed Group Co., Ltd., Wuhan, China, 430206) was utilized for genomic background analysis of progeny line K608R2123. Standard quality control was implemented (removal of non-polymorphic loci and heterozygous parental markers), yielding N valid markers. The background recovery rate was calculated according to the following formula:

R(%) = (A + 0.5H)/N × 100,(1)

where a denotes the number of homozygous recurrent parent genotypes, and H represents the number of heterozygous genotypes.

### 4.4. Bacterial Blight Resistance Analysis

*Xoo* inoculation was performed at the maximum tillering stage (55–60 days after transplanting). Six purified *Xoo* races were selected: PXO86, PXO112, PXO99, PXO145, PXO280, and XOO4. A bacterial suspension of 3 × 10^8^ CFU·mL^−1^ (with an optical density at OD600 nm about 0.5) was prepared for inoculation. The leaf-cutting inoculation was performed within one hour [39]. Inoculation was conducted by clipping leaf tips (~2 cm) with scissors dipped in bacterial suspension, with 3–5 leaves cut per plant. Disease resistance was evaluated 15 days post-inoculation when lesion expansion stabilized and clear symptoms were observable. Lesion lengths were measured using rulers, by researchers blinded to the plant genotypes. For each *Xoo* race, ten independent plants were inoculated as biological replicates (*n* = 10).

### 4.5. Characterization of Agro-Morphological Traits

Agronomic traits were assessed at physiological maturity (125–130 days after transplanting), with each treatment consisting of 3 biological replicates. The following agronomic trait variables were recorded for the pyramided lines K608R2123 and parents (K608R, K608R21, and K608R23): plant height (cm), panicle length (cm), panicles/plant, panicle weight (g), no. of grains/panicle, 1000-seed weight (g), and single plant yield (g). Variation coefficients (CV) of the indicators were calculated using the following formula:

(2) $$\mathrm{CV}=\sigma /(\bar{x})$$

### 4.6. Data Analysis

Statistics and reproducible quantitative data, including 3–10 biological and at least three technical replicates, are presented in the means + standard deviations. Means of two samples were compared using Student’s two-tailed t-tests. Analysis of variance (one-way ANOVA) was conducted using GraphPad Prism 9.0.0 software with default parameters. Significant differences were determined by one-way ANOVA or Student’s *t*-test: * *p* < 0.05, ** *p* < 0.01,*** *p* < 0.001.

## 5. Conclusions

In summary, this study establishes a robust FMAS-based strategy for efficiently pyramiding BB resistance genes in rice. The K608R2123 line, combining *Xa21* and *Xa23*, exhibits broad-spectrum resistance (including against the Hainan-prevalent XOO4 race) without compromising agronomic performance, offering a promising solution for sustainable BB management in hybrid rice production. Its suitability for southern China’s “nanfan” system further enhances its practical value. As a distinct, uniform, and stable variety, it meets key requirements for deployment. Our findings highlight the importance of precision breeding tools in developing resilient crop varieties, contributing to global food security in the face of evolving pathogenic threats.

## Figures and Tables

**Table 1:** Recombination frequency of F_2_.

Number of F_2_ Populations	Genotype *Xa21*^+−^/*Xa23*^++^	Genotype *Xa21*^++^/*Xa23*^+−^	Recombination Frequency (%)
624	3	2	0.84

**Table 2:** Variation coefficients (CVs) of agronomic traits.

Agronomic Trait	Variation Coefficient (CV)
Plant height (cm)	2.244
Panicles/plant	5.908
Panicle length (cm)	3.553
Panicle weight (g)	7.778
No. of grains/panicle	2.952
Seed-setting rate (%)	3.014
1000-seed weight (g)	3.199
Single plant yield (g)	7.471

## Data Availability

The data presented in this study are available on request from the corresponding author.

## Supplementary Materials

The following supporting information can be downloaded at: https://www.mdpi.com/article/10.3390/plants14142107/s1, Figure S1: Analysis of disease resistance of parents K608R, K608R23, K608R21, and pyramided plants K608R2123.

## Abbreviations

The following abbreviations are used in this manuscript:

BB	bacterial blight
*Xoo*	*Xanthomonas oryzae* pv. *oryzae*
FMAS	functional marker-assisted selection
R	resistance
FM	functional marker
SNP	single nucleotide polymorphism
CMS	cytoplasmic male sterile
LRR	leucine-rich repeat
MAS	marker-assisted selection
CV	coefficient of variation
