# Supplementary figures and images for: Efficient Marker-Assisted Pyramiding of Xa21 and Xa23 Genes into Elite Rice Restorer Lines Confers Broad-Spectrum Resistance to Bacterial Blight
Source: Plants (Basel). 2025 Jul 9;14(14):2107. doi: 10.3390/plants14142107 (PMC12298770; doi:10.3390/plants14142107)

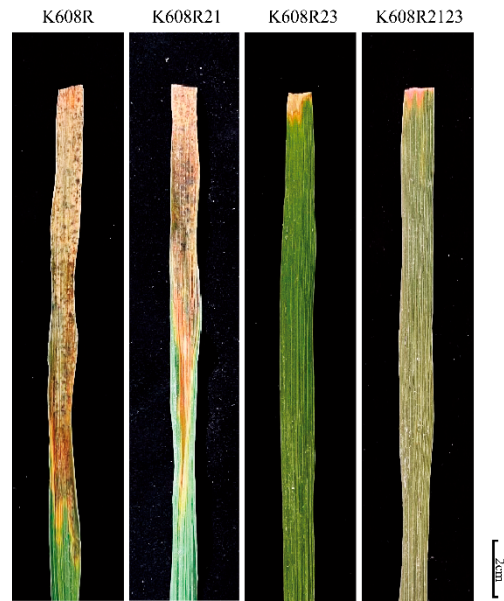

**Figure S1** Analysis of disease resistance of parents K608R, K608R23, K608R21, and pyramided plants K608R2123. Scale=2cm.

Supplement: Supplementary file 1 [file plants-14-02107-s001.zip › plants-3705899-supplementary.pdf]
